# Supplementary figures and images for: MreC and MreD balance the interaction between the elongasome proteins PBP2 and RodA
Source: PLoS Genet. 2020 Dec 28;16(12):e1009276. doi: 10.1371/journal.pgen.1009276 (PMC7793260; doi:10.1371/journal.pgen.1009276)

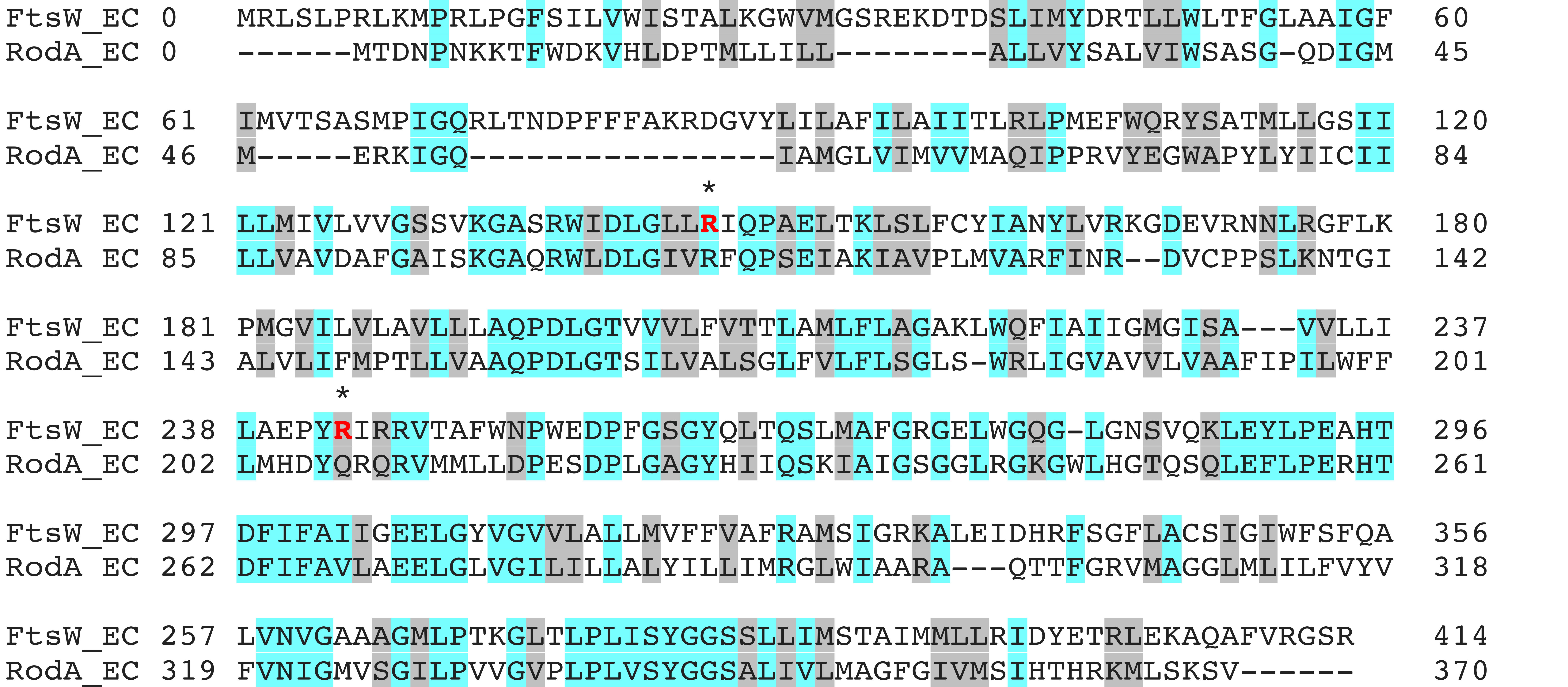

Supplement: S1 Fig — The two inactive RodA mutants were constructed based on the previous functional studies of FtsW [1, 2] (bold red and labeled with *). The protein sequence alignment was generated with the online tool Multiple Sequence Alignment (MUSCLE). Shadow colors indicate the average BLOSUM62 score of the paired residues: light blue> = 3 (identical amino acids), light gray> = 2 (similar amino acids), no color (different amino acids). (TIF) [file pgen.1009276.s001.tif]

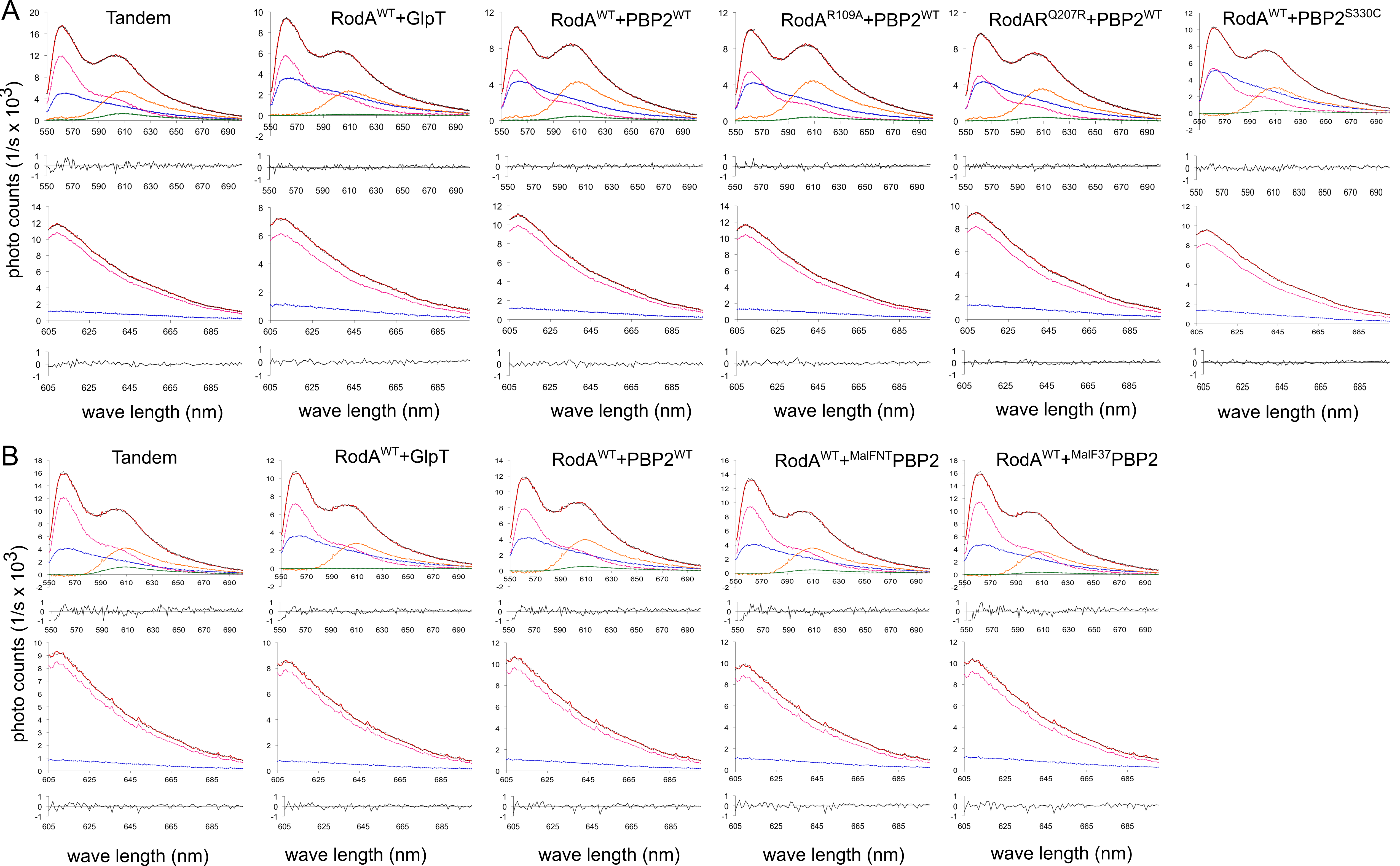

Supplement: S2 Fig — A. Inactive RodA and PBP2 variants shown in Fig 2B. B. Domain swap variants of PBP2 shown in Fig 3C. LMC500 cells expressing each FRET pair were grown in Gb4 medium to steady state at 28°C and further cultured in the presence of 15 μM IPTG for 2 mass doublings. FRET pairs are listed above the spectra. For each pair the upper panel contains the measured spectrum excited at 538 nm in black dots, the calculated spectrum (in red) and its unmixed components; blue is background, magenta is mKO, orange is mCherry and green is the sensitized emission. The middle panel is the measured spectrum of mCherry excited at 590 nm in black dots, the calculated spectrum (in red) and its unmixed components; blue is background and magenta is mCherry. The bottom panel shows the residuals of the measured and calculated spectrum. (TIF) [file pgen.1009276.s002.tif]

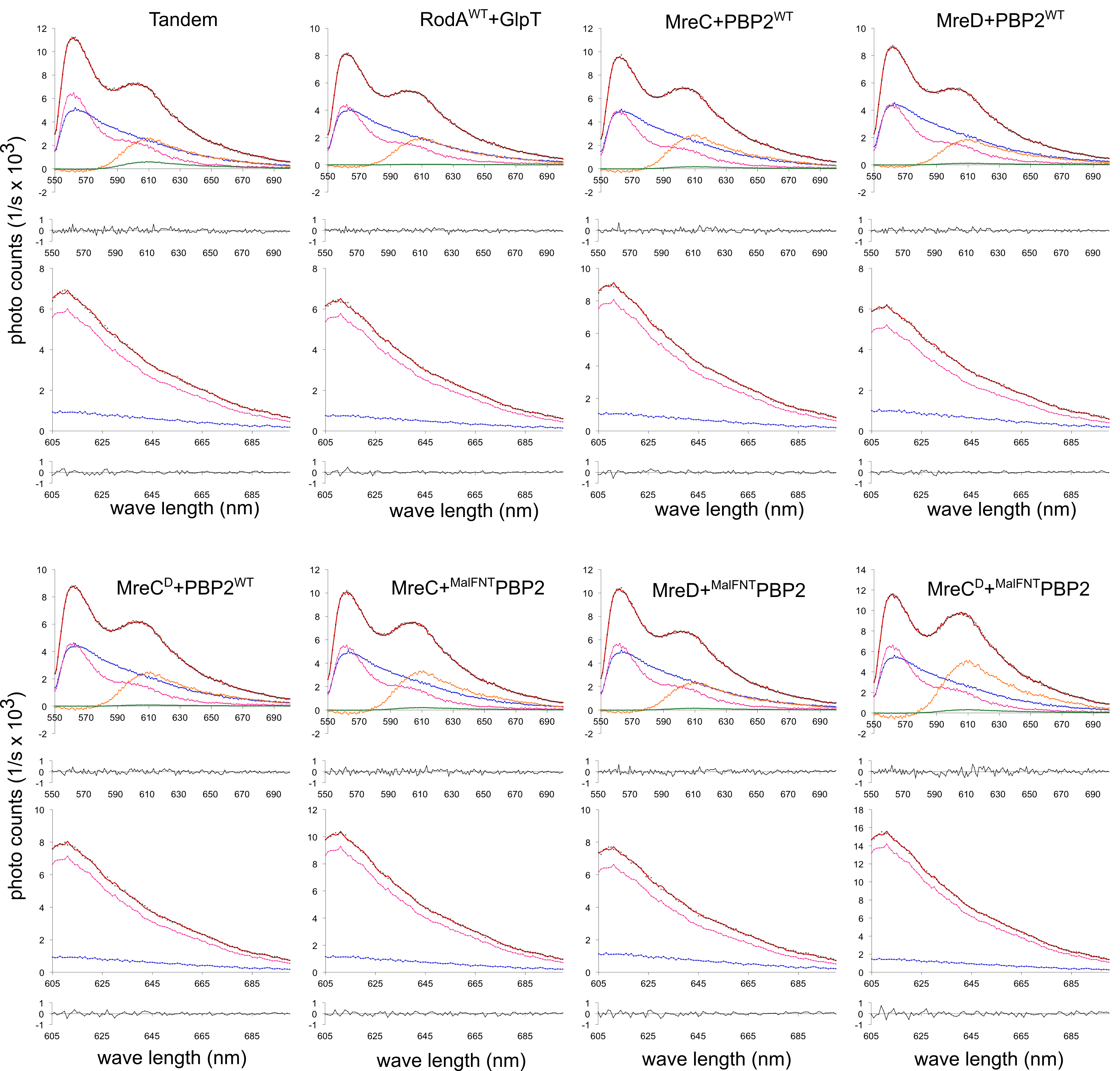

Supplement: S3 Fig — LMC500 strain expressing each FRET pair was grown in Gb4 medium to steady state at 28°C and induced with 15 μM IPTG for 2 mass doublings. FRET pairs are listed above the spectra. MreCD, MreC and MreD were expressed from one plasmid, and MreC was fused with mCherry while MreD was not fused. Panels as in S2 Fig. (TIF) [file pgen.1009276.s003.tif]

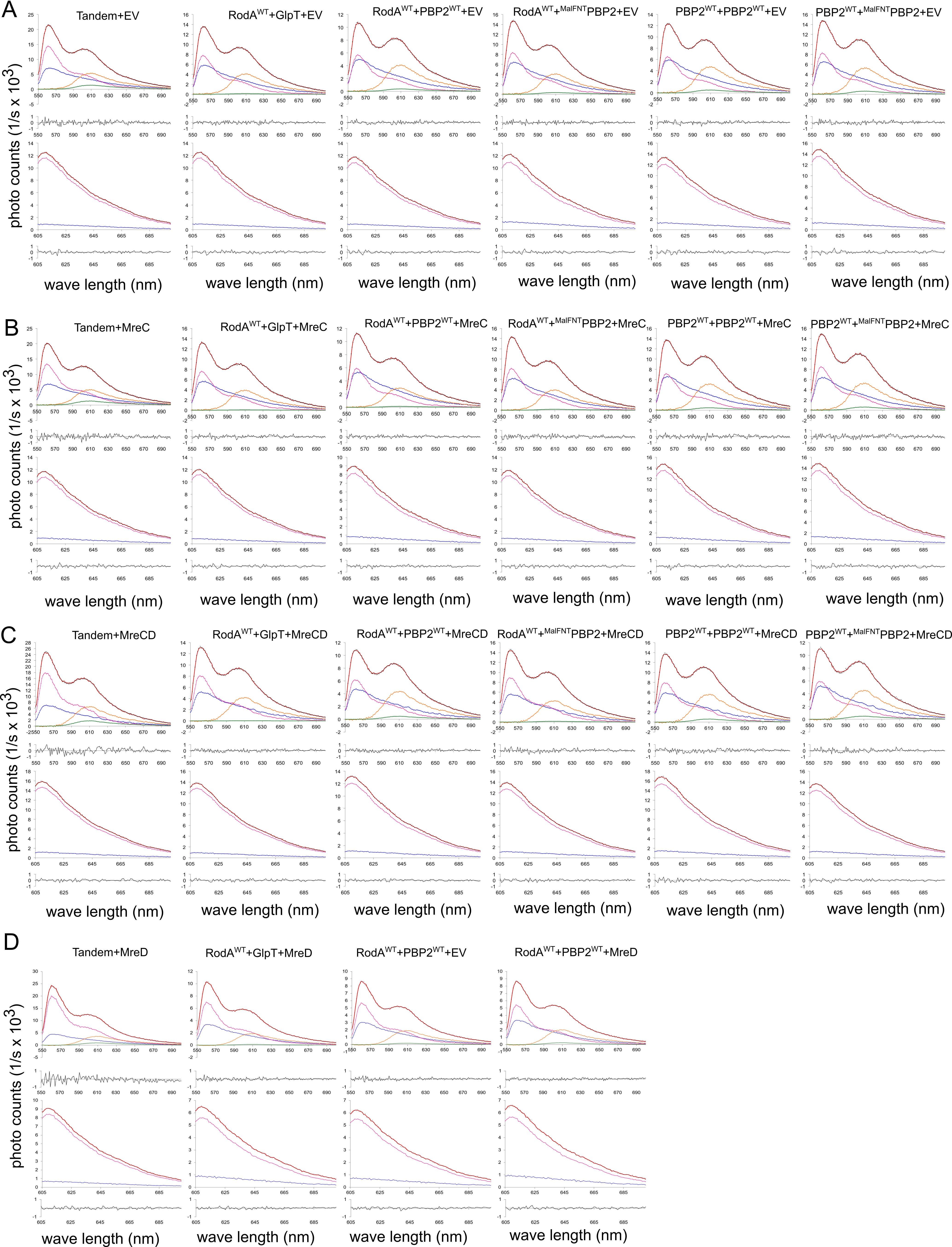

Supplement: S4 Fig — FRET pairs are listed above the spectra. A, B, C and D. Unmixing data of FRET experiments in the presence of the third: empty vector (EV), or MreC (expressing MreC alone from the third plasmid under a Ptrcdown promoter), MreCD (expressing MreCD together from the third plasmid under a Ptrcdown promoter), or MreD alone from the third plasmid, respectively. Panels as in S2 Fig. (TIF) [file pgen.1009276.s004.tif]

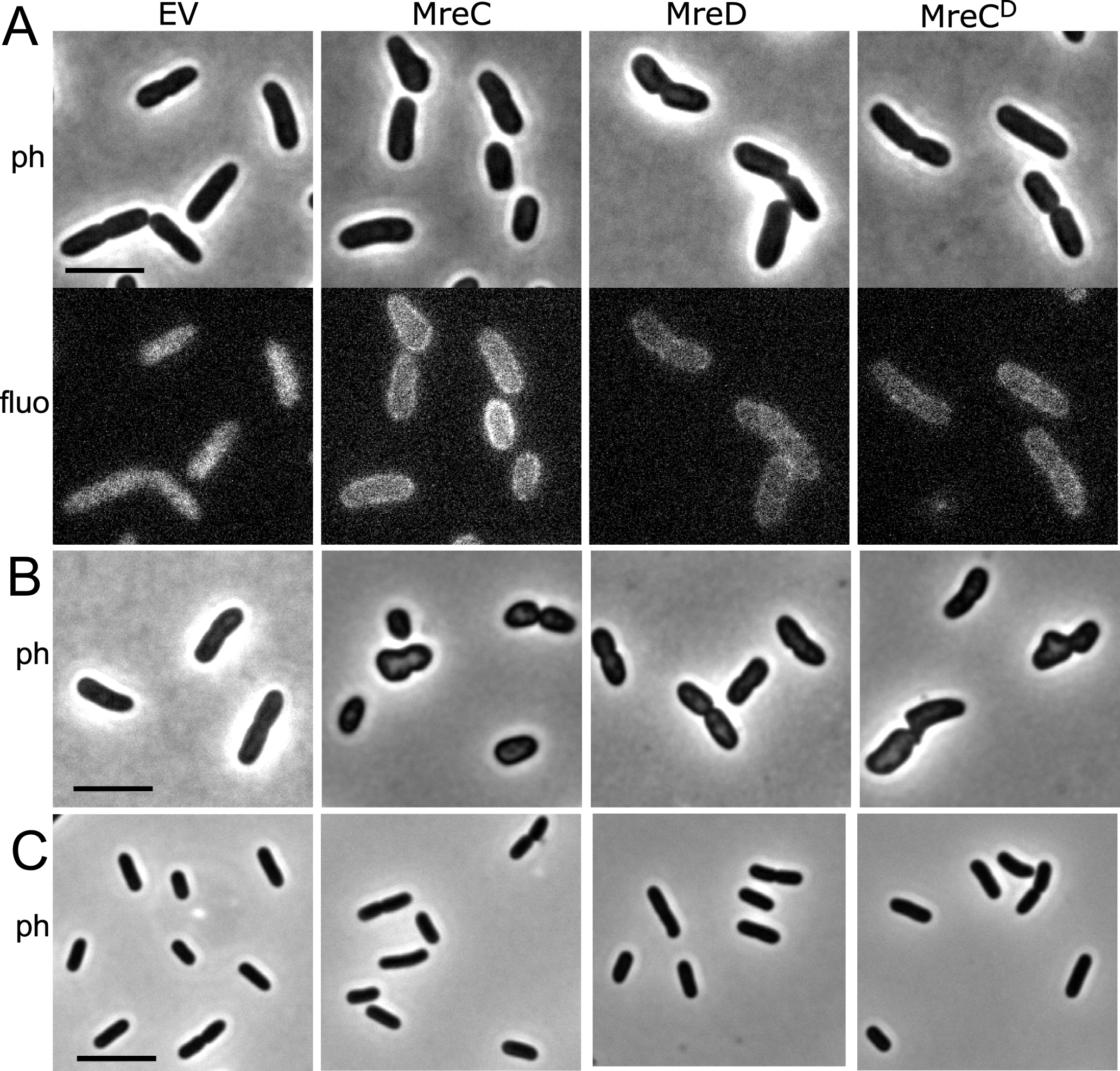

Supplement: S5 Fig — A. Phase contrast (upper panels) and fluorescent (bottom panels) images of the morphology and FP-protein localization, respectively, of LMC500 cells expressing mCherry (control), mCherry-MreC, mCherry-MreD or mCherry-MreCD. MreCD were expressed from one plasmid and MreC was fused with mCherry, while MreD was non-fused. Cells were growth in LB medium at 37°C. B and C. Images of the morphology of LMC500 cells with empty vector, expressing MreC, MreD or MreCD grown in LB at 37°C or in minimal glucose medium (GB4) at 28°C. Expression was induced for two mass doublings with 15 μM IPTG. Scale bar equals 5 μm. (TIF) [file pgen.1009276.s005.tif]

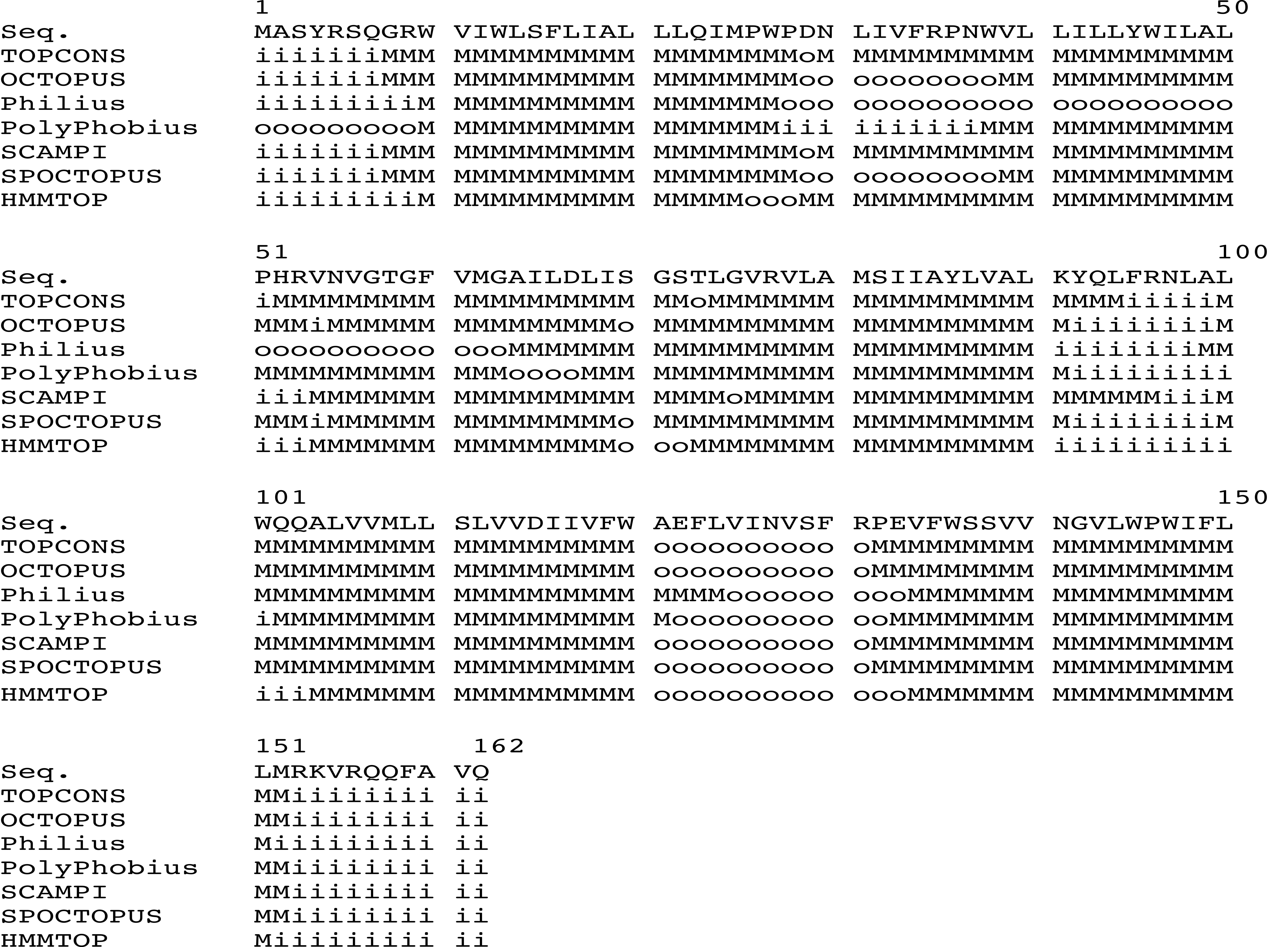

Supplement: S6 Fig — Numbers: MreD amino acid position. i: in the cytoplasm. o: in the periplasm. M: transmembrane sequences. (TIF) [file pgen.1009276.s006.tif]

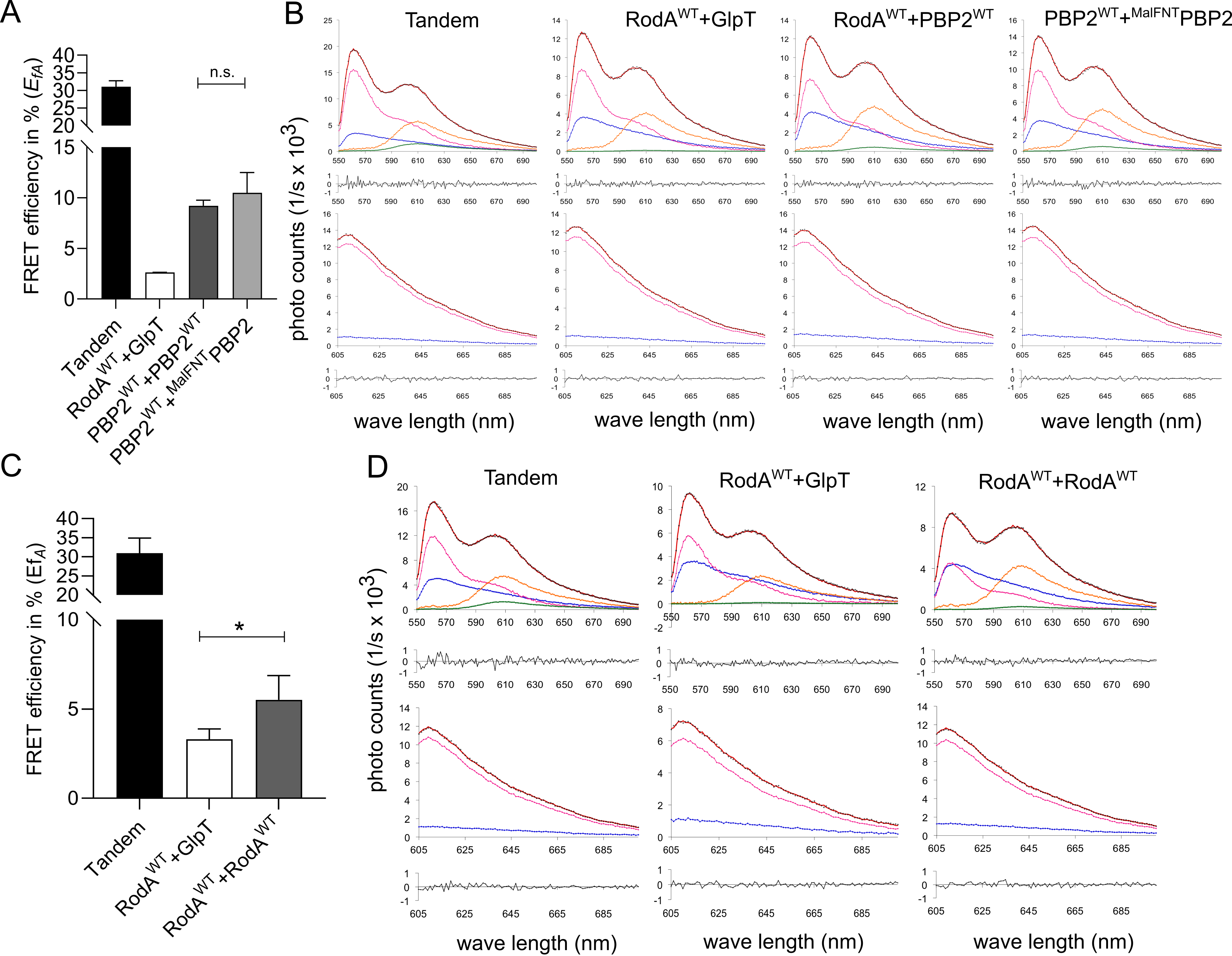

Supplement: S7 Fig — LMC500 cells expressing each FRET pair were grown in Gb4 medium to steady state at 28°C and further cultured in the presence of 15 μM IPTG for 2 mass doublings. A. Acceptor FRET efficiency (EfA) between PBP2WT and PBP2WT calculated from the spectral FRET measurements. B. Overview of the spectral unmixing data of all the FRET pairs of self-interacting PBP2 and its variants. C and D. Calculated acceptor FRET efficiency (EfA) and spectral unmixing data of RodA self-interaction. FRET pairs are listed above the spectra. Panels as in S2 Fig. P value determined with Student’s t-test (n.s.: not significant; (*: p<0.05)). (TIF) [file pgen.1009276.s007.tif]

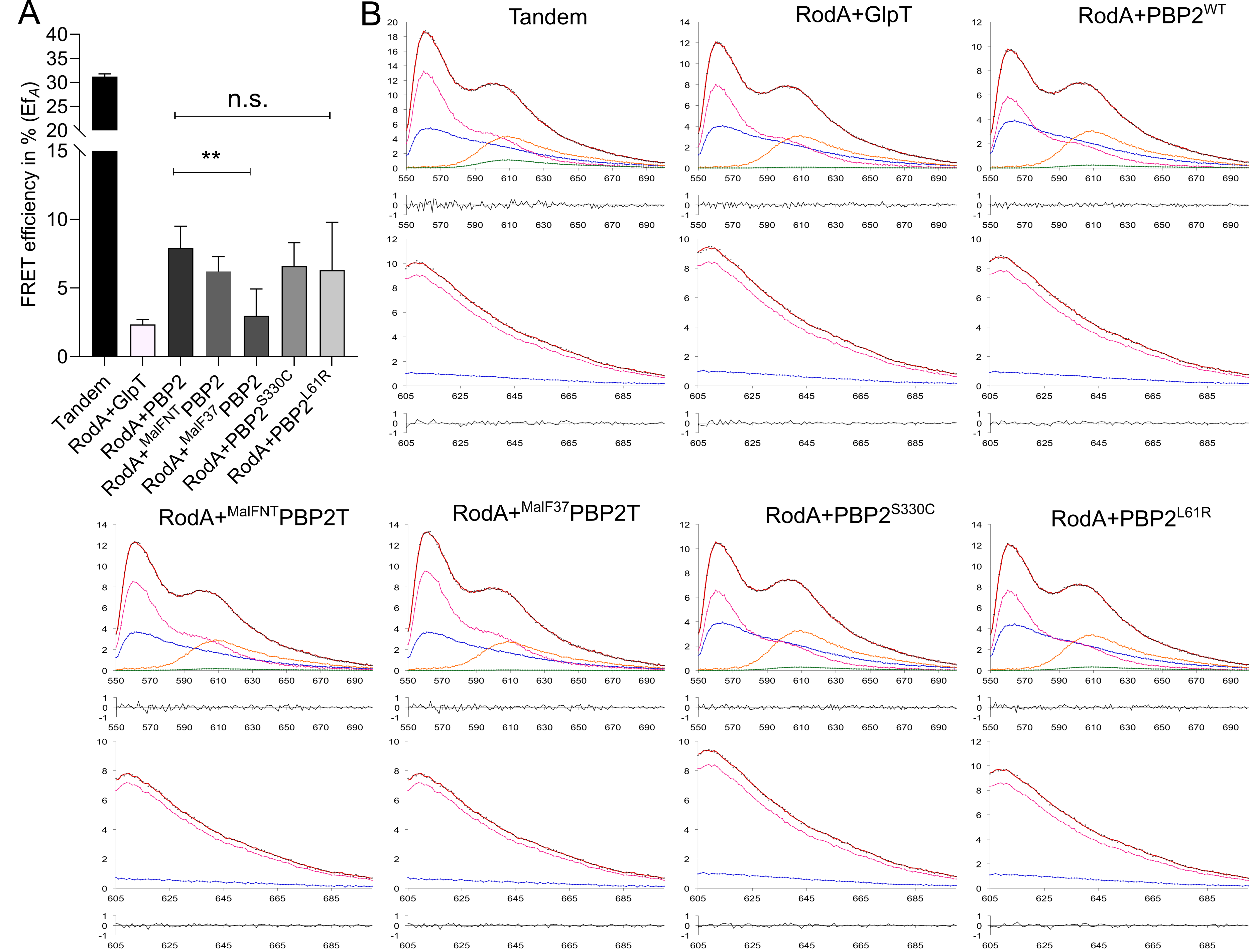

Supplement: S8 Fig — LMC500 cells expressing each FRET pair were grown in Gb4 medium to steady state at 28°C and further cultured in the presence of 15 μM IPTG and treated with mecillinam (2 μg·mL-1) for 2 mass doublings. a. Acceptor FRET efficiency (EfA) calculated from the spectral FRET measurements. P value determined with Student’s t-test (**: p<0.01). b. Overview of the unmixing data of all the FRET samples showed in S8A Fig. FRET pairs are listed above the spectra. Panels as in S2 Fig. (TIF) [file pgen.1009276.s008.tif]

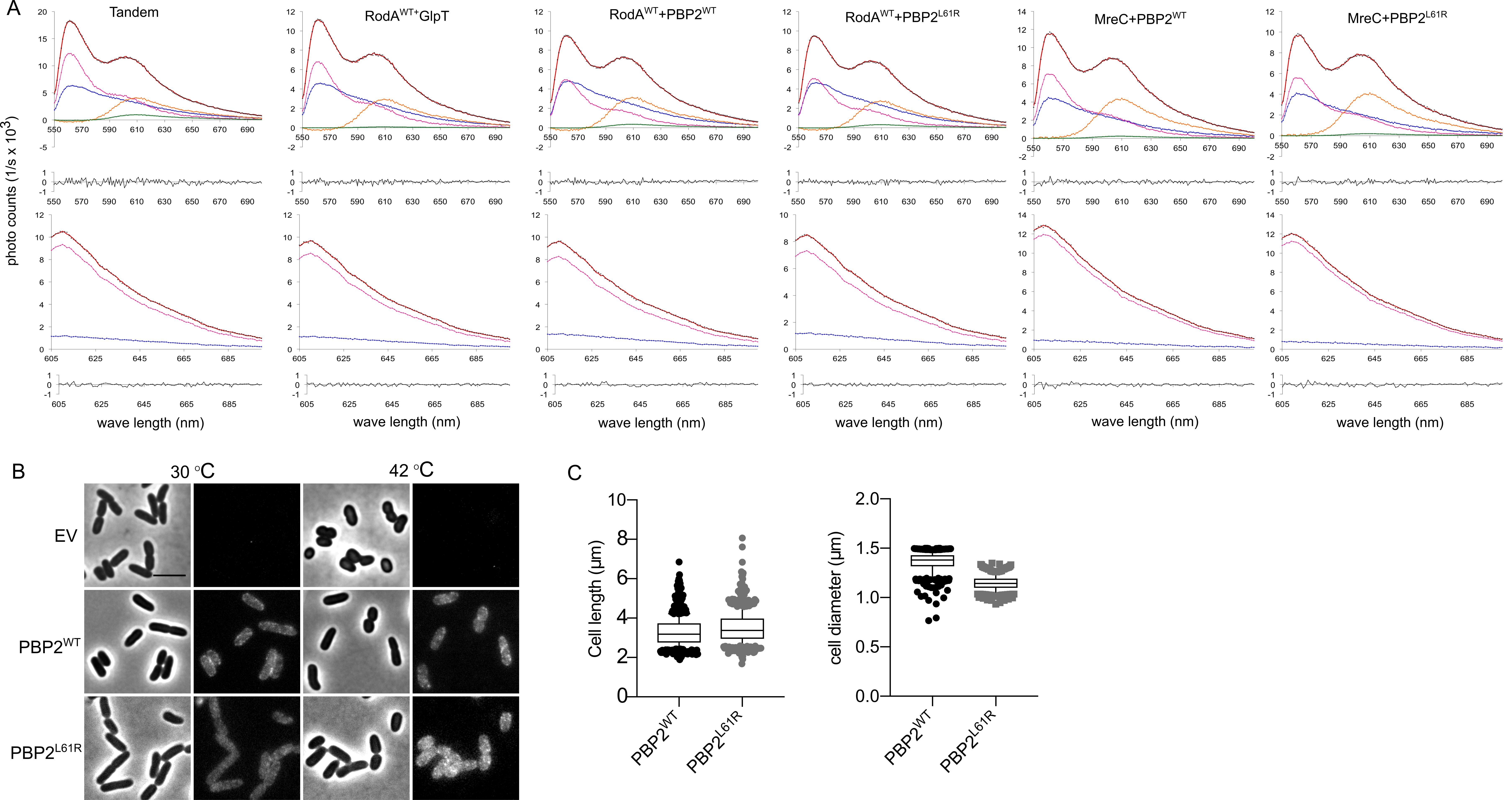

Supplement: S9 Fig — A. Overview of the spectral unmixing data of all the FRET groups between hyperactive PBP2L61R and its interaction partners that are listed in Fig 5B. LMC500 cells expressing each FRET pair were grown in Gb4 medium to steady state at 28°C and further cultured in the presence of 15 μM IPTG for 2 mass doublings. FRET pairs are listed above the graphs. Panels as in S2 Fig. B. Phase contrast and fluorescence images of the complementation of cells harboring a PBP2 temperature sensitive allele with PBP2WT or hyperactive PBP2L61R. PBP2 temperature sensitive strain LMC582 was transformed with empty vector (EV), mKO-PBP2WT plasmid, or mKO-PBP2L61R plasmid and grown in LB medium at 30°C (left panels) and 42°C (right panels), respectively, and the expression of PBP2 versions was induced with 15 μM IPTG for 2 mass doublings. Scale bar equals 5 μm. C. Expression of the hyperactive PBP2L61R results into (slightly) longer and thinner cells. LMC500 strain was transformed with plasmid expressing either mKO-PBP2WT or mKO-PBP2L61R, grown in LB at 37°C and further cultured in the presence of 15 μM IPTG for 2 mass doublings. Cells were fixed and imaged by microscopy. Over 1000 cells were measured for statistical analysis. (TIF) [file pgen.1009276.s009.tif]

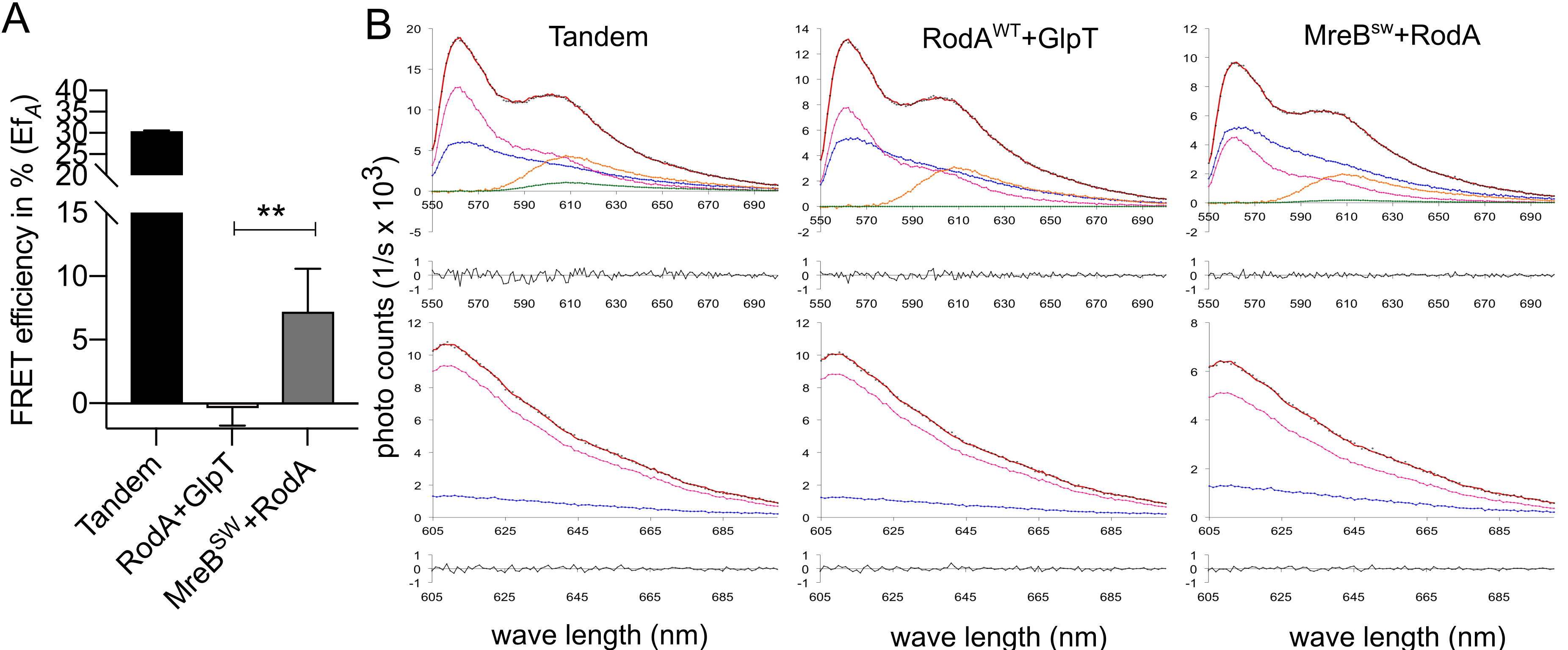

Supplement: S10 Fig — LMC500 cells expressing each FRET pair were grown in Gb4 medium to steady state at 28°C and further cultured in the presence of 15 μM IPTG for 2 mass doublings. A. Acceptor FRET efficiency (EfA) calculated from the spectral FRET measurements. P value determined with Student’s t-test (**: p<0.01). B. Overview of the unmixing data of all the FRET samples showed in S10A Fig. FRET pairs are listed above the spectra. Panels as in S2 Fig. (TIF) [file pgen.1009276.s010.tif]
