# Supplementary material for: MreC and MreD balance the interaction between the elongasome proteins PBP2 and RodA
Source: PLoS Genet. 2020 Dec 28;16(12):e1009276. doi: 10.1371/journal.pgen.1009276 (PMC7793260; doi:10.1371/journal.pgen.1009276)
Supplement: S1 Table — (DOCX) [file pgen.1009276.s011.docx]

**S1 Table.** **Muropeptide composition of LMC500 strains carrying no plasmid or different expression plasmids.**

| Muropeptide^2^ or  feature | Percent peak area (%)^1^ | | | | | |  |
| --- | --- | --- | --- | --- | --- | --- | --- |
|  | LMC500 | LMC500 mKO | LMC500 mKO-PBP2 | LMC500 mKO-PBP2^L61R^ | | LMC500 mKO-GlpT | |
| Tri | 5.0 ± 0.2 | 4.9 ± 0.0 | 5.0 ± 0.4 | 4.0 ± 0.2 | 4.7 ± 0.1 | | |
| TetraGly | 1.0 ± 0.0 | 1.0 ± 0.0 | 1.5 ± 0.0 | 1.2 ± 0.1 | 1.1 ± 0.1 | | |
| Tetra | 41.5 ±0.2 | 40.8 ± 0.0 | 41.0 ± 0.2 | 43.1 ± 1.3 | 42.8 ± 0.2 | | |
| Di | 1.4 ± 0.0 | 1.6 ± 0.1 | 1.3 ± 0.1 | 1.2 ± 0.0 | 1.4 ± 0.0 | | |
| TriLysArg | 3.7 ± 0.4 | 3.4 ± 0.2 | 3.5 ± 0.5 | 3.0 ± 1.2 | 3.4 ± 0.5 | | |
| TetraTri(Dap) | 1.4 ± 0.2 | 1.5 ± 0.0 | 2.1 ± 0.2 | 2.4 ± 0.3 | 1.8 ± 0.2 | | |
| TetraTetraGly | 0.2 ± 0.1 | 0.4 ± 0.0 | 0.2 ± 0.1 | 0.2 ± 0.1 | 0.2 ± 0.1 | | |
| TetraTri | 3.1 ± 0.3 | 2.9 ± 0.2 | 3.0 ± 0.1 | 2.2 ± 0.0 | 2.9 ± 0.1 | | |
| TetraTetra | 33.6 ± 0.8 | 33.6 ± 0.4 | 32.4 ± 2.1 | 35.1 ± 2.2 | 33.8 ± 0.3 | | |
| TetraAnh | 0.9 ± 0.0 | 1.0 ± 0.0 | 1.0 ± 0.0 | 0.7 ± 0.0 | 1.0 ± 0.0 | | |
| TetraTri Lys Arg | 1.5 ± 0.1 | 1.7 ± 0.1 | 1.5 ± 0.2 | 1.2 ± 0.2 | 1.6 ± 0.1 | | |
| TetraTetraTetra | 3.3 ± 0.0 | 3.4 ± 0.0 | 3.4 ± 0.0 | 2.9 ± 0.0 | 3.4 ± 0.0 | | |
| TeraTetraAnh I | 1.1 ± 0.0 | 1.1 ± 0.0 | 1.2 ± 0.1 | 1.0 ± 0.0 | 1.1 ± 0.0 | | |
| TeraTetraAnh II | 0.9 ± 0.0 | 1.0 ± 0.0 | 1.0 ± 0.0 | 0.7 ± 0.0 | 0.9 ± 0.0 | | |
| TetraTetraTetra Anh | 1.1 ± 0.0 | 1.2 ± 0.0 | 1.3 ± 0.0 | 0.8 ± 0.0 | 1.1 ± 0.0 | | |
| Sum of all known | 99.6 ± 0.3 | 99.4 ± 0.1 | 99.2 ± 0.4 | 99.6 ± 0.4 | 100 ± 0.0 | | |

^1^ values are mean ± variation of two biological replicates.

^2^ muropeptide names according to Glauner, 1988(1).

**References**

1. Glauner B, Holtje J V., Schwarz U. The composition of the murein of Escherichia coli. J Biol Chem. 1988;263(21):10088–95.
