# Supplementary material for: MreC and MreD balance the interaction between the elongasome proteins PBP2 and RodA
Source: PLoS Genet. 2020 Dec 28;16(12):e1009276. doi: 10.1371/journal.pgen.1009276 (PMC7793260; doi:10.1371/journal.pgen.1009276)
Supplement: S2 Table — (DOCX) [file pgen.1009276.s012.docx]

**S2 Table.** **Strains and plasmids used in this study.**

|  | Relevant properties | References |
| --- | --- | --- |
| ***Strains*** |  |  |
| DH5α | *supE44 ΔlacU169 (Ф80lacZΔM15) hsdR17 recA1 endA1 gyrA96 thi-1 relA1* | Lab stock |
| LMC500  (*MC4100 lysA*) | *F^-^ araD139 Δ(argF-lac)U169 deoC1 flbB5301 ptsF25 rbsR relA1 rpsL150 lysA1* | (1) |
| LMC882 | *his,purB,proA,thi,lacY,rpsL,rodA(Ts)-52,zbe::Tn10* | (2) |
| LMC582 | *LMC500 pbpa137 (ts)* | (3) |
| **Plasmids** |  |  |
| pTHV037 | pTRC99A with a weakened P_trcdown_ promoter. pBR322 ori, ampicillin resistance | (4) |
| pSAV057 | pTRC99A with a weakened P_trcdown_ promoter. p15A ori, chloramphenicol resistance | (4) |
| pSG4K5 | The empty third plasmid used for three-plasmids FRET. pSC101 ori, Kanamycin resistance | (5) |
| pSAV047 | pTHV037 expressing with mcherry gene | (4) |
| pSAV058 | pSAV057 expressing mKO gene | (4) |
| pSAV050 | pSAV057 expressing mCherry-mKO tandem gene | (4) |
| pSAV047-RodA | pTHV037 expressing mCh-RodA fusion gene linked with five codons | (4) |
| pWA003 | pTHV037 expressing mCh-PBP2 fusion gene | (4) |
| pWA004 | pSAV057 expressing mKO-PBP2 fusion gene | (4) |
| pRP058 | pTHV037 expressing mCh-MreB sandwich fusion gene | (4) |
| pXL28 | pSAV057 expressing mNG-(GGS)_2_-GlpT fusion gene | (6) |
| pXL29 | pSAV057 expressing mKO-(GGS)_2_-GlpT fusion gene | This study |
| pXL36 | pTHV037 expressing mCh-RodA^R109A^ fusion gene | This study |
| pXL40 | pTHV037 expressing mCh-RodA^Q207R^ fusion gene | This study |
| pXL44 | pSAV057 expressing RodA^R109A^ gene | This study |
| pXL48 | pSAV057 expressing RodA^Q207R^ gene | This study |
| pXL56 | pSAV057 expressing mKO-RodA fusion gene linked with five codons | This study |
| pXL63 | pSAV057 expressing RodA^WT^ gene | This study |
| pXL148 | pSAV057 expressing mKO-^MalFNT^PBP2 fusion gene | This study |
| pXL149 | pSAV057 expressingmKO-^MalF37^PBP2 fusion gene | This study |
| pXL158 | pSAV057 expressing mKO-PBP2^S330C^ fusion gene | This study |
| pXL159 | pSAV057 expressing mKO-PBP2^L61R^ fusion gene | This study |
| pXL165 | pTHV037 expressing mCh-MreC fusion gene that linked with 5 codons | This study |
| pXL166 | pTHV037 expressing mCh-MreCD genes. MreC is fused to mCh with 5 codons. MreD is not fused | This study |
| pXL167 | pSG4K5-derivative that expressing MreC genes under the P_trcdwon_ promoter. Used for three plasmids FRET | This study |
| pXL168 | pSG4K5-derivative that expressing MreCD genes under the P_trcdwon_ promoter. Used for three plasmids FRET | This study |
| pXL169 | pTHV037 expressing mCh-MreD fusion gene that linked with 5 codons | This study |

**References**

1. Taschner PE, Huls PG, Pas E, Woldringh CL. 1988. Division behavior and shape changes in isogenic *ftsZ, ftsQ, ftsA, pbpB*, and *ftsE* cell division mutants of *Escherichia* *coli* during temperature shift experiments. J Bacteriol 170:1533–1540.

2. Matsuzawa H, Hayakawa K, Sato T, Imahori K. 1973. Characterization and genetic analysis of a mutant of *Escherichia coli* K-12 with rounded morphology. J Bacteriol 115:436–442.

3. van der Ploeg R, Verheul J, Vischer NOE, Alexeeva S, Hoogendoorn E, Postma M, Banzhaf M, Vollmer W, Blaauwen den T. 2013. Colocalization and interaction between elongasome and divisome during a preparative cell division phase in *Escherichia coli*. Mol Microbiol 87:1074–1087. doi: 10.1111/mmi.12150.

4. van der Ploeg R, Goudelis ST, Blaauwen den T. 2015. Validation of FRET Assay for the Screening of Growth Inhibitors of *Escherichia coli* Reveals Elongasome Assembly Dynamics. IJMS 16:17637–17654. doi: 10.3390/ijms160817637.

5. Standage-Beier K, Zhang Q, Wang X. 2015. Targeted Large-Scale Deletion of Bacterial Genomes Using CRISPR-Nickases. ACS Synth Biol 4:1217–1225. doi: 10.1021/acssynbio.5b00132.

6. Liu X, Meiresonne NY, Bouhss A, Blaauwen den T. 2018. FtsW activity and lipid II synthesis are required for recruitment of MurJ to midcell during cell division in *Escherichia coli.* Mol Microbiol. 109:855-884. doi: 10.1111/mmi.14104.
