# Supplementary material for: MreC and MreD balance the interaction between the elongasome proteins PBP2 and RodA
Source: PLoS Genet. 2020 Dec 28;16(12):e1009276. doi: 10.1371/journal.pgen.1009276 (PMC7793260; doi:10.1371/journal.pgen.1009276)
Supplement: S3 Table — (DOCX) [file pgen.1009276.s013.docx]

**S3 Table.** **Primers used in this study.**

| **Primer** | **Sequence 5’-3’** | **Description** |
| --- | --- | --- |
| priXL59 | GCGCGAATTCATGACGGATAATCCGAATAAA | EcoRI-RodA-F |
| priXL60 | GCGCAAGCTTTTACACGCTTTTCGACAACAT | HindIII-RodA-R |
| priXL61 | CGACGGCTGAAAGGCAACAATACCTAGGTCCAGCC | RodA-R109A-R |
| priXL69 | ATGCATGATTACCGTCGCCAGCGC | RodA-Q207R-F |
| priXL70 | GCGCTGGCGACGGTAATCATGCAT | RodA-Q207R-R |
| priXL146 | TGCTGCCAGTGGTGCTTTTGCAT | GA-57bone-F |
| priXL147 | CTTGAGTCCAACCCGGAAAGACA | GA-57bone-R |
| priXL258 | TCGCTTTGCCACCAATGTTTCTTTTTAATGACATCCATGTTGTTGTTGAATTCGGAATG | GA-mKO-NNN-MalF-R |
| priXL259 | ATTAAAAAGAAACATTGGTGGCAAAGCGACGCGCTGAAAGCGCTGGTCGCCTTTTTGGG | GA-PBP2-del-NT-F |
| priXL260 | GCTGGTGGGTTACCTTGTTGTTTTAATGTATGGCCAGCGCTTTACCGACTACCAGACC | GA-PBP2-del42-F |
| priXL261 | ATGGATGTCATTAAAAAGAAAC | MalF-F |
| priXL263 | CTGGCCATACATTAAAACAAC | MalF-NTTMH1-R |
| priXL274 | GTTTATCCGCCGGCGTGTACAGTTAAACC | PBP2-S330C-F |
| priXL275 | GGTTTAACTGTACACGCCGGCGGATAAACCCCCT | PBP2-S330C-R |
| priXL276 | CGCATTAAGCGGGTGCCGATCGCGCC | PBP2-L61R-F |
| priXL277 | TGGGCGCGATCGGCACCCGCTTAATGCGGT | PBP2-L61R-R |
| priXL282 | CCCGAATTCAACAACAACATGAAGCCAATTTTTAGCCGTGG | EcoRI-MreCD-F |
| priXL283 | GCGCAAGCTTTTATTGCACTGCAAACTGCTGAC | HindIII-MreCD-R |
| priXL284 | TGAGCGGATAACAATTTCACACAGGAAACAGACCATGAAGCCAATTTTTAGCCGTGG | MreCD-GA-F |
| priXL286 | GCGCAAGCTTTATTGCCCTCCCGGCGCACG | HindIII-MreC-R |
| priXL299 | GCGCGAATTCAACAACAACGTGGCGAGCTATCGTAGCCA | EcoRI-NNN-MreD-F |
| priXL294 | CTAGATAATTGGAGACCGAGCT | GA-pXL167-168-F1 |
| priXL295 | CGAATTCGAGTCACTAAGGGCT | GA-pXL167-168-R1 |
| priXL296 | GTAATTAGTTAGTTAGCCCTTAGTGACTCGAATTCGCTGGCAAATATTCTGAAATGAGC | GA-pXL167-168-F2 |
| priXL297 | ACATCCTAGAGAGACCAGCTCGGTCTCCAATTATCTAGCTATTGCCCTCCCGGCGCACG | GA-pXL167-R |
| priXL298 | ATCCTAGAGAGACCAGCTCGGTCTCCAATTATCTAGTTATTGCACTGCAAACTGCTGAC | GA-pXL168-R |
| PP15 | GGTCTGTTTCCTGTGTGAAATTGTTATCCGC | p_trcdown_-R |
