# Supplementary material for: MreC and MreD balance the interaction between the elongasome proteins PBP2 and RodA
Source: PLoS Genet. 2020 Dec 28;16(12):e1009276. doi: 10.1371/journal.pgen.1009276 (PMC7793260; doi:10.1371/journal.pgen.1009276)
Supplement: Attachment — Submitted filename: Answers to comments of Plos Genetics reviewers .pdf [file pgen.1009276.s017.pdf]

We thank all reviewers for their constructive comments and questions. We hope that we have been able to solve all raised matters to satisfaction.

Reviewer #1: This paper uses a FRET approach to interrogate interactions of proteins in the sidewall peptidoglycan (PG) elongosome of Escherichia coli. In particular, the work probes the interactions between the Class B PBP2 transpeptidase (TP) and its SEDS glycosyltransferase (GT) RodA that are mediated by the regulatory proteins MreC and MreD. This study builds on in-cell FRET approaches established and validated by Prof. den Baauwen's laboratory over the last decade. The work incorporates ideas from structural studies of the MreC-PBP2 interaction and mutant protein constructs discovered as suppressors or null active site variants in previous studies. It also determines the PG crosslinking composition and glycan chain lengths in the mutants. Together, a model emerges for the role of MreC and MreD in elongosome regulation. The model proposes that MreC switches the PBP-RodA activities to an "on" or activated state, whereas MreD returns the functions to the off state. This model is analogous to the one for regulation of septal PG synthesis, which is also mediated by an off/on mechanism.

The sophisticated FRET and PG composition experiments were rigorously performed, and the data are complete with appropriate statistical analyses. The data here are new and will be of considerable importance and interest to the field. This impactful paper is logical and very well written. There are a couple of major points that need to be addressed and/or explained to strengthen certain interpretations and conclusions. These points primarily concern experimental issues that could impact cellular physiology, and hence the interpretation of the results and the working model.

Major points

1. Results and Discussion. Please present data and/or comments about the relative expression levels of the fluorescent-protein constructs relative to wild-type. All of the constructs are expressed from low-copy number plasmids at limiting inducer levels as worked out in previous studies.

A. Nevertheless, to interpret the physiological relevance of some of these results, it becomes important to know how much combined fluorescent-tag and native untagged protein are being expressed, at least for PBP2, RodA, MreC, and MreD. How close are the protein amounts to the WT levels, and how might increased cellular abundance influence the interpretations? Is the relative 2:1 abundance of MreC to MreD, which is invoked in the Discussion as regulatory, maintained in the constructs? Are the protein mutant constructs expressed at same level as the non-mutant constructs?

B. It seems like polyclonal antibodies against each of these long-studied E. coli proteins should be available for determinations of relative amounts by quantitative western blotting.

C. At any rate, this topic should be discussed more in the paper, because it affects the interpretations of the FRET experiments and the dominant-negative phenotypes, which can be skewed by relative overexpression.

Answer to 1A. In all FRET experiments, more tagged material is produced than the endogenous expression of the proteins. Of course endogenous expression levels would be ideal. Unfortunately, these proteins come in very low numbers of a few hundred (or even less) at endogenous expression level, which is too low to measure any reliable FRET. Based on our previous experiments, we express about 2000 molecules from plasmid. The results are therefore related to the proteins that are expressed from plasmid not to the endogenous elongasome complex as such. Consequently, if we want to know what the effect is of elongasome proteins they also have to be expressed from plasmid (hence the three plasmid FRET). All FRET proteins were expressed from the low copy number plasmids, and were induced with same amount of IPTG for 2 mass doubling time, which yield comparable expression for wild type and mutants proteins according to the measured FRET spectra (see supplementary figures). About the 2:1 ratio question, please, see our reply to comment #3

Answer to 1B. Unfortunately, good polyclonal antibodies against these *E. coli* proteins are not available. We have much experience with antibodies and like them very much (see our previous papers), but it is not easy to obtain high quality antibodies against *E. coli* proteins as most animals are contaminated with *E. coli* already. The market is not big enough for commercial enterprises to make antibodies against *E. coli* proteins.

Therefore, we have tried to visualize the amount of protein produced by other means. We have made a westernblot of our FRET cultures, to detect the expression of RodA and PBP2 proteins (reviewer Fig. 1a). mCherry fused RodA proteins were detected with antibody against mCherry. Since RodA is a multi-membrane-spanning protein that cannot be boiled for western blotting, it is not known where to expect the bands. However comparing the mCherry-RodA with mCherry only and with the mCherry-mKO tandem, it is clear that the very fat band is the complete fusion. Double bands might reflect proteins that are synthesized but not yet translocated across the periplasm. The amount of fusion protein of the various FRET pairs seems to be similar (reviewer Fig 1a). If any degradation is observed it seems to be very similar for the various samples. These results indicate that mCherry-RodA constructs are expressed at a similar level in our FRET experiments, which is in agreement with the similarity of the mCherry emission units in the spectra of the FRET measurement in the supplementary figures.

Similarly, we tried to establish whether mKO-PBP2 variants were equally expressed. We do not have antibodies against PBP2, therefore, we bought antibodies against mKO. Unfortunately, these antibodies did not recognize mKO and the company has stopped selling the antibody. Next we tried to visualize PBP2 with the fluorescent penicillin variant Bocillin-FL. The mCherry-RodA ampicillin resistant plasmid expresses beta-lactamase, which is also able to degrade Bocillin FL. Therefore we expressed mKO-PBP2 without RodA in the strain used for the FRET experiment and tried to visualize the protein with Bocillin-FL. We could only make PBP2 very weakly visible. We reasoned that PBP2 needs perhaps its partner RodA to bind Bocillin-FL efficiently. Consequently, we had to design a plasmid identical to the plasmid expressing mCherry-RodA but now with Spectinomycin resistance and combine this plasmid with that of mKO-PBP2. We repeated the FRET experiment to be sure that the different plasmid did not affect the FRET data and detected mCherry-RodA from this experiment with anti mCherry and mKO-PBP2 with the Bocillin-FL probe. The Bocillin signal is very weak in our hands as we lack equipment that is able to measure the Bocillin FL efficiently and we could not travel to better equipment due to the Corona restrictions. We observed a band at

approximately 90 kDa (Reviewer Fig. 1b) that corresponds to PBP1A and mKO-PBP2, we also observed a usually a more dense band at the position of PBP2 comparing with the empty vector. This could indicate some breakdown of the construct either in living cells or during the Bocillin FL binding procedure. The ratio of the PBP2 mKO-PBP2 bands seems the same for all constructs indicating that difference in FRET values are not caused by differences in synthesis or degradation. The labelling procedure requires incubation time and washing to remove the excess of Bocilin FL. For the FRET experiment the cells are grown and fixed in a shaking water bath, which is a completely different condition as needed for the Bocillin Fl labelling experiment. When mKO-PBP2 variants are expressed form plasmid without the mCherry-RodA partner, all variants show similar membrane localization with very little cytosolic signal (reviewer Fig. 1c). (Due to spectral overlap mCherry and mKO cannot be imaged separately when both are present in the cell.) Therefore the amount of mKO in the spectra likely correlates well with the amount of mKO-PBP2 expressed

All these results suggest that in our FRET experiments, the mutants proteins were expressed at the same level as wild type, and the FRET efficiency differences should reflect the changes in the protein interaction.

Answer 1C. We measure only FRET changes form plasmid expressed proteins and include for all conditions always the wild type combination and positive and negative controls to be able to assess the quality of the experiment. The intensity of the spectra allows quantification of the amount of protein produced (see supplementary figures).

2. Results and Discussion. L. 348 mentions a severe “tag interference” effect. In the other FRET experiments using two tagged proteins, were any milder tag effects observed that affected cell growth and/or morphology? If so, please indicate these cases and whether other tag effects might affect certain interpretations.

We thank the reviewer for this interesting question, but we think the loss of mKO signal in this FRET experiment was not due to the “tag interference”. The mKO-PBP2^{L61R} and untagged PBP2^{L61R} have no detectable morphological difference. In our two plasmids FRET experiment, this mutant could be co-expressed with mCh-RodA for interaction study without losing the mKO signal. Therefore, we are confident that mKO did not affected the behaviour of this mutant. Given the fact that PBP2^{L61R} (either tagged or untagged) causes some changes in the morphology of *E. coli*, and MreC/MreD proteins alter the RodA-PBP2 interaction, we assumed these combinations were toxic to cells, resulting in the loss of the mKO signal.

3. L. 209 and L. 443. The method in the paper by Li et. al. to determine absolute proteins synthesis rates is powerful, but makes the huge assumption that all proteins are equally stable, implying that the rates reflect the steady-state amounts. The 2-fold difference in relative amounts of MreC and MreD becomes important later for the model. Has the relative steady-state amounts of these two proteins (or other E. coli elongosome proteins) been determined directly by western blotting for cells grown in the media used? This corroborative information would considerably strengthen some central aspects of the model proposed here. It would also indicate any cleavage of tags off of proteins.

We agree with the reviewer that this 2:1 ration might not reflect the real concentration of the proteins in the cell. However, our results indicate that the effect of MreC and MreD on the RodA-PBP2 subcomplex is opposite and that their balanced presence is therefore important, and it does not have to be a 2:1 ratio. Based on our results and the actual similarities between elongasome and divisome components, we hypothesized these complexes could possible share some formally similar regulation mechanisms, and thus proposed our model. The accurate ratio for the MreC and MreD competition in the elongasome, also the ratio of FtsN-FtsQLB in the divisome, require some future studies. We have changed our discussion accordingly.

4. L. 243. Was the MreC overexpression phenotype dependent on the tag or was it also observed for overexpression of wild-type MreC? Please clarify.

We have expressed tagged and untagged MreC (new Fig. S5) and observed similar morphological changes. Expression of tagged or untagged MreD slightly increased the diameter of the cells. While we noticed that when expressing untagged MreCD proteins together in rich medium, the morphology of some cells was not as good restored as for tagged MreCD. This might due to the expression difference between tagged and untagged proteins that differed the balance of MreCD proteins. Indeed, when grown in minimal medium, which produces less protein copies, the expressed proteins hardly affected the morphology of the cells. (new Fig. S5).

5. L. 314 and 331. In L. 314, it seems confusing that metcillinam causes a reduction, but active site changes don't. You might add ("see below"), so this point is not left hanging. In L. 331, is there any structural data for a separate antibiotic binding site? If so, please site.

We thank reviewer for this suggestion. To our knowledge, there is no published structural data for a separate antibiotic binding site. Mecillinam causes a reduction in all the RodA-PBP2 FRET pairs, including the PBP2 domain swap variants and inactive variant. Although S330C cannot covalently bind mecillinam, it likely is still able to bind mecillinam noncovalently (Houba-Hérin, N. et al., (1985) Mol Genet 201: 499-504 and personal observations for PBPs with active site serine mutants). Because the number of repeats for the mecillinam experiments was rather low we did additional repeats and have used these data to calculate new Ef_A values (see highlights in Table 1). The new data further confirmed the old data and did not change the conclusions. We have changed this part in our manuscript as suggested.

6. L. 366 and L394. If PBP2(L61R) is activated for TP activity, wouldn't you expect increased reaction with mecillinam resulting in hypersensitivity? Also, the pbp2(L61R) mutations suppresses delta-mreC mutations and allows growth. That is, the PBP2(I61R) functions fully in the complete absence of MreC, which according to the model is required for stimulation of TP activity. These data do not seem to quite fit with the model. Please clarify.

Cells expressing PBP2^{L61R} are more sensitive to mecillinam than expressing PBP2^{WT}, which indicates a weakened PG for the mutant, not necessarily a higher affinity for mecillinam. A weaker PG can be caused by a reduction in peptide cross bridges or an increase in glycan strand length. The latter was found for the mutant, suggesting that this mutant is hyperactive because it activates RodA, but is not different with respect to its TPase activity comparing with PBP2^{WT}. RodA-PBP2^{L61R} interaction was not different from the WT pair, indicating they are likely in the same conformational state as we discussed. Substrate binding in the form of mecillinam does affect the interaction between the two proteins, which results again in similar Ef_A values for mutant and wild type. This suggest that the affinity of WT PBP2 and PBP2^{L61R} for mecillinam is likely the same.

Moreover, although the hyperactive mutant PBP2^{L61R} could allow growth of ΔMreC and ΔMreCD cells, the morphology was poorly restored, which strongly indicating that this mutant is not fully functional without MreCD proteins.

Interestingly, the hyperactive RodA^{A234T}, which enhances the glycosyltransferase activity of RodA but likely not doing anything with PBP2 transpeptidase activity, can also supress the MreC defects, although it cannot allow the growth in the absence of MreCD proteins. One possible explanation could be that the enhance GTase of RodA^{A234T} is not as strongly enhanced as by PBP2^{L61R}, as shown by the Patricia D. A. Rohs et al. study (2018).

Together, these results give an indication that PBP2^{L61R} might still need MreC for its proper TPase activity, the enhanced GTase activity is possibly contributing more to the suppression of MreC defects.

7. L. 451. The results of Li et al. were obtained for three different growth conditions, which seemed to change the MreC and MreD amounts, although the 2:1 ratio is maintained. It seems that some additional trigger mechanism needs to be added that works in conjunction with MreC/MreD, since their relative amounts are maintained. Please clarify further.

In general the concentration of proteins is quite constant in *E. coli* growing in different media as can be seen from the data of Li et al.,. For many protein complexes, the critical ratio between their component proteins are also constant for different growth conditions according to this study, including the divisome proteins FtsQLB-FtsN that we are comparing to. It is clear that maintaining these ratios is very important for *E.coli*. This does not exclude additional trigger mechanism for the elongasome and divisome.

Minor points

8. L. 123. Please change to: “RodA oppositely...”
This had been changed.
9. L. 199. For clarity, please change to: “...sufficient for the wild-type interaction...”
This had been changed.
10. Table 1, 2, etc. The footnotes might mention that P values for comparisons are shown in

the figures.
This footnote had been added to Table 1 and Table 2.
11. L. 180 and L. 309. Please change to: “As shown”.
This had been changed.
12. L. 284. Please delete” “wild type PBP2” (i.e., should read “with MreC and MreD”).
This had been changed as suggested.

13. L. 285 and elsewhere. “wild-type” as an adjective should be hyphenated.
This had been changed.
14. L. 285 and 288. Please change: “Fig. 4d” to “Fig. 4c”.
This had been changed as suggested.
15. L. 290: Please change the last “dash” between PBP2 and RodA to a “+” because it is referring to the FRET experiment (i.e., “value of RodA+...). Also, please check the labels in the figures where some dashes should be changed to pluses.
These “dash” labels in the text and tables had been changed to “+” to match the labels in figures.
16. L. 294. Please consider changing to: “plays a role”.
This has been changed as suggested.
17. L. 338. Please change to: “If this were the case,”
This had been changed.
18. L. 347. Please change: “side” to “site”.
This had been changed.
19 L. 432. Please change to: “on PBP2 activity is formally similar”.
This had been changed.
20. L. 544. Please change to: “pH 7.2”.
This had been changed.
21. L. 566. Please change to: “7000 rpm”.
This had been changed.

Reviewer #2: The manuscript presents very solid evidences on interaction between RodA (an integral membrane protein with glycosil transferase activity) and PBP2 (performing transpeptidase activity). Besides, the manuscript presents very interesting data on regulation of the biosynthetic machinery by the MreC and MreD system. Overall the manuscript presents a very nice framework to understand regulation of bacterial cell wall biosynthesis in elongasome. Figures and text are clear, and well supported by the previously reported literature on this topic as well as in agreement with recent structural results. The manuscript would be of general interest for a broad audience, specially for microbiologists.

We thank the reviewer for his/hers positive remarks and agree happily.

Reviewer #3: The manuscript by Liu et al. addresses an important and challenging question in bacterial morphogenesis: how PG synthesis is regulated in the context of the elongasome, the multiprotein complex that orchestrates lateral cell wall growth and maintains rod shape.

Understanding the roles of individual components of the elongasome has not been trivial because loss of function mutations are not particularly informative and the system cannot yet be reconstituted in vitro. Liu et al. attempted to bypass these limitations by using FRET to interrogate interactions and potential alternative conformations of elongasome components in vivo. They focused on the interaction between PBP2 and RodA and arrived at a model in which MreC and MreD have antagonistic effects in the regulation of the PBP2-RodA enzymatic core of the elongasome, with MreC being an activator and MreD an inhibitor of PBP2. FRET seems like a good choice to learn more about elongasome regulation, the Den Blaauwen group is experienced with the technique, the manuscript is technically sound and contains plenty of data. However, I am concerned that the main conclusions of the paper are not well supported by the data, as explained below.

Specific comments:

1) The key hypothesis of the paper, that the reduction in PBP2-RodA FRET efficiency produced by MreC reflects an activating conformational change of PBP2, does not seem consistent with other observations. **One important inconsistency is with the effects of MreC and MreD overexpression on elongasome function, as inferred from the increase in cell diameter (Fig. 4e). The increase in diameter suggests that both MreC and MreD overexpression make the elongasome less functional. Thus, the FRET reduction produced by MreC overexpression correlates with a less functional elongasome, not the reverse.** Likewise, the concomitant overxpression of MreC and MreD rescues both FRET efficiency and elongasome activity, as measured by cell diameter (Figs. 4d and 4e). An important missing experiment in this series is the effect of MreD overexpression alone in PBP2-RodA FRET efficiency. What if FRET efficiency decreases upon MreD overexpression? Even if it does not, the authors should be mindful of alternative explanations for their data, which are probably many, including unexpected ways in which perturbing the stoichiometry of the elongasome will affect its organization.

We agree with the reviewer that overexpression of MreC and MreD affects the diameter of the cells indicating that the function of the elongasome is disturbed. This can especially be seen when the proteins are expressed in rich medium (37°C) grown cells, where induction with 15 μM IPTG leads to higher expression levels than in minimal medium (28°C) grown cells (new Fig. S5). The FRET experiments are performed in minimal medium grown cells. Under these circumstances expression of MreC or MreD or both hardly affects the morphology of the cells (new Fig S5). Expression of PBP2 + RodA does not affect the morphology of the cells. Therefore, we assume that this complex is not active in the absence of the other elongasome proteins and can readily exchange with elongasome proteins without causing any harm. As soon as we express non-functional partners such as the S330C variant, they will affect the function of the elongasome and change the morphology of the cells. Expression of only MreC or only MreD or the wrong ratio of MreCD have negative effects on the elongasome, presumably because they cause a disbalance. Evidence provided by Rohs et al. (2018) point to an activation of PBP2 by MreC. Together, it seems to us more likely that our changed FRET values reflect an activation and not an inactivation by MreC.

We did repeat a number of FRET experiment and added additional experiments as suggested by other comments, however, we were unable to add the suggested FRET experiment in present of MreD because of the impossibility to do many experiments due to the corona closure of the lab. Because of the lack of strong effects on the morphology of the cells by MreD, we do not expect an effect of MreD on the interaction between RodA and PBP2. Also, the three-plasmid FRET is more difficult and time consuming requiring at least two weeks for one nice repeat. Showing significantly that a proteins has no effect would require at least 4 repeats. As the lab was closed for 6 month we gave priority to repeating the FRET experiments with mecillinam.

Another important inconsistency is with the behavior of the L61R mutation. This mutation has no effect on the PBP2-RodA FRET efficiency despite having all the phenotypic and structural features expected of a constitutively active PBP2 protein. This should certainly have prompted more tests of the hypothesis that lower FRET is indeed reporting the PBP2 on state. Instead, the authors take their FRET correlation at face value and create an "ad hoc" explanation for the unexpected behavior of the L61R mutation.

Ideally, the authors should try to provide other types of experimental evidence that tie the reduced FRET with the PBP2 on state. I understand this is not easy, however, the FRET information, as it stands, is just too vague to support the paper´s conclusions.

We thank the review for this interesting question, but we would like to humbly express our disagreements with the reviewer about our data on hyperactive mutant $PBP2^{L61R}$, as we do not think this is an inconsistency, and we have showed additional experiments and data to support our hypothesis

First of all, this mutant indeed showed phenotypic features that are different with wild-type PBP2, but this is not necessarily to be only the expectation of a constitutively active PBP2 mutant. As shown in study of Patricia D. A. Rohs et al. (2018) the $RodA^{A234T}$ hyperactive mutant showed similar features as $PBP2^{L61R}$. And to our knowledge, there is no structural evidence available to show that this mutant has a constitutively active conformation. Actually, the study of Patricia D. A. Rohs et al. (2018) hypothesized that this mutant is constitutively in the active state, which mimics MreC activation.

Secondly, after we observed these unexpected FRET results of the $PBP2^{L61R}$ mutant, we indeed carried our further experiments before finalizing our conclusion, as showed in the manuscript. In agreement with the previously reported data in the study of Patricia D. A. Rohs et al. (2018), this mutant indeed results into longer and thinner cells, and increases the *E. coli* resistance to A22. However, we additionally found that this mutant increases the mecillinam sensitivity, which likely indicates a weaker peptidoglycan layer. This further prompted us to check carefully using biochemistry tools to analysis the peptidoglycan composition. The analysis results strongly suggest that $PBP2^{L61R}$ stimulates the GTase activity (of RodA) without changing its TPase activity.

Moreover, before making our final conclusion about the hyperactive mutant PBP2^{L61R}, we also investigated why expressing it changed the dynamics of MreB (Rohs et al., 2018). We found a direct interaction between MreB and RodA in our FRET experiment. The enhanced RodA function by PBP2^{L61R} could then possible be an explanation for the changes in MreB dynamics.

All these results finally give us the confidence to conclude that thePBP2^{L61R} hyperactive mutant is behaving similarly as wild-type PBP2 with respect to its TPase activity, but possibly stimulates the GTase activity of RodA

2) How can the authors conclude that two proteins interact directly from FRET alone? This seems particularly complicated when the efficiencies are low as in the case of the pair MreD-PBP2. What distance does that translate into? 10 nm seems like enough to allow even indirect interactions to produce a FRET signal, in particular when the FP tags are connected to the interacting proteins via flexible linkers, as seems to be the case here.

Our *in vivo* FRET is very well developed over the last decade. The potential interacting candidates are mildly overexpressed from two low-copy plasmids. We had detected many protein interactions with FRET in previous and current studies, and always set many references and controls for the measurements and calculations, and the overexpression is supposed to sufficient to avoid the influence from the native proteins, so the detected FRET signals are principally from the direct interaction detecting protein pairs. Over years, we had used different protein combinations (proteins from very different complexes, have very different functions and localizations) that are very likely not interacting directly, and stabilized the bottom line of direct interaction around 2%-3% FRET signal of mCh-mKO FRET system. After many biological repeats and statistical analysis, the potential interacting protein pairs that yield stable and obvious higher FRET efficiency will be considered as directly interacting.

3) The analysis of the interaction surface between PBP2 and RodA would be strengthened by testing point mutations predicted by Sjodt et al. 2018.

In this study, we focused more on the protein PBP2 and its variants, and showed how changes in PBP2 can influence its interaction with its partner protein RodA. That was why we did not include more mutants of RodA in this study. We agree with the reviewer that RodA and PBP2 interaction could be strengthened by testing more RodA mutants, and we indeed have a manuscript in preparation that focus on RodA and its variants. According to the studies on RodA homologous, we had constructed over 20 mutants of RodA in that story, and carried our detailed investigations on RodA function and its interaction with elongasome proteins. But we prefer to keep these as a separate story, and not include this information in this manuscript.

4) Why in the 3 plasmid experiments the FRET efficiency of RodA-PBP2 goes down to 8%, with the empty vector? The authors claim that this is a general effect of the burden of maintaining 3 plasmids but no such reduction is seen with the positive control.

The positive control is the tandem fusion of the donor and acceptor fluorophores, which makes them always together at fixed distance. That is why the FRET efficiency of the positive control is not changed over different conditions and systems (two plasmids-FRET or 3 plasmids-FRET). This also indicates that our FRET system is reliable and stable to measure the interactions between proteins.

We concluded that the reduction of the FRET efficiency in 3 plasmids-FRET is the general effect of the burden of maintain 3 plasmids, as we observed that the mass doubling time during FRET increased from about 110 min of two-plasmids-FRET to about 150 min of three-plasmids-FRET, indicating a global influence on cellular activities. The important point is that we are comparing the FRET efficiency changes always in the same system, the observation of reduction from 8% to 4% in the three-plasmid FRET reflects the RodA-PBP2 interaction changes upon the presence of different third proteins. We cannot actually compare the FRET efficiencies between two-plasmid FRET and three-plasmid FRET directly.

5) The manuscript has many typographical errors, some examples below:
l. 191 – Increase instead of decrease
This had been changed.
l. 205 – respectively43?
This had been deleted.
l. 210 – remove by
This had been changed.
l. 222 – remove we
This had been changed.
l. 298 – our results have
This had been changed.
