# Supplementary material for: MreC and MreD balance the interaction between the elongasome proteins PBP2 and RodA
Source: PLoS Genet. 2020 Dec 28;16(12):e1009276. doi: 10.1371/journal.pgen.1009276 (PMC7793260; doi:10.1371/journal.pgen.1009276)
Supplement: Attachment — Submitted filename: PGENETICS-D-19-01848R1 reply to comments of reviewers R2.pdf [file pgen.1009276.s018.pdf]

PGENETICS-D-19-01848R1

Reviewer's Responses to Questions

Comments to the Authors:
Please note here if the review is uploaded as an attachment.

Reviewer #1: The authors have provided thorough, careful, and thoughtful responses to all of the reviewers' previous comments. Their responses are notable for their deep consideration and detail and address all of the previous issues, within the limits of this experimental system. The result is a highly rigorous study using this form of FRET analysis that leads to an interesting new model for the functions of MreC and MreD in E. coli. My view is that the revised version is an important paper that presents an interesting model that certainly will be further tested by additional approaches in E. coli and other bacterial models.

Thank you very much for your enthusiasm.

Reviewer #2: I appreciate authors effort in doing this revised version and I maintain my original point of view about the quality of the present manuscript. I would like, however, indicate some points that would need to be included/discussed in the final version of the manuscript:

1. Concerning the discussion on mecillinam effect on PBP2. There are, at least, two structural precedents of antibiotic binding at allosteric sites far from the active site. The first one is the structure of pneumococcal PBP2X in complex with cefuroxime (Gordon et al, J. Mol. Biol. 2000, 299, 477– 485) and the second one is the structure of PBP2a from MRSA in complex with ceftaroline (Otero et al, Proc. Natl. Acad. Sci. U. S. A. 2013, 110, 16808–16813). To my knowledge, there is a single example of an antibiotic non-covalently bound to the active site of a PBP, the 3D structure of pneumococcal PBP2X in complex with cefepime (Bernardo-Garcia et al ACS Chem. Biol. 2018, 13, 3, 694–702).

Thank you for this very useful information, which was not at the forefront of our thoughts.

We have added this information to the paper as follows:

"suggesting that the mutant can still interact with the antibiotic at its active site. Alternatively, mecillinam binds to a second (allosteric) site that activates the protein as has been reported for some β-lactams and PBPs {Otero:fe, Gordon:2000dc, BernardoGarcia:2018ba}. High concentrations of aztreonam that do not inhibit PBP2 cause an increase in binding of the fluorescent β-lactam Bocillin FL {Kocaoglu:2015dw}, suggesting that an allosteric site might be present in PBP2. Two recently published crystal structures of *E. coli* PBP2 in the presence of avibactams show only binding to the active site {Levy:2019kv} indicating that at least these antibiotics do not bind to an allosteric site at the used concentrations. The change in FRET efficiency upon binding of mecillinam, which is measured in the cytoplasm, suggests conformational changes in the complex that are transmitted via the transmembrane areas. Based on the crystal structures of PBPs with allosteric sites, such a site is expected to be at the interface between V-shaped MreC-binding domain of PBP2 and the globular β-lactam binding domain {Otero:fe, Gordon:2000dc}. An allosteric site would make sense for a TPase that has to bind two peptide side chains to be able to make the crosslink. Allosteric activation of the TPase reaction would make sure that the protein is only active in the presence of a disaccharide peptide that is in the process of being transglycosylated by RodA. Our observation that mecillinam binding cause a reduction in FRET similar to the presence of MreC, even of the active site mutant S330C, might plead for the presence of an allosteric site in PBP2."

We also added some information on this in the discussion (see yellow text in the manuscript).

2. In the manuscript (and also in some of the Reviewers comments) there is an important body of discussion about the potential role of PBP2 L61R mutant. During the time for elaboration of this revised version there has been important news, from the structural point of view, about this system. The crystal structure of PBP2 from E. coli (EcPBP2) has been reported alone and in complex with some antibiotics (Levy et al, J. Medicinal Chem. 2019, 62, 9, 4742–4754) and the crystal structure of the PBP2:RodA complex from Thermus thermophilus (TtPBP2:RodA) has been also reported (Sjodt et al, Nature Microbiology 2020, volume 5, pages813–820). It is curious this recent information is not mentioned in the revised manuscript as it deals directly with the discussion and results here presented by a different technique. I think the results here presented should be discussed in the

light of the new structural and functional results recently reported for the same complex. The 3D structure of TtPBP2:RodA presents the PBP2 lying onto the RodA protein, very close to the membrane and far from the PG in an inactive configuration for transpeptidation. In this sense, Sjodt et al 2020 propose the interaction with MreC is required in order the PBP2 to reach the PG.

Our apologies for not including the paper of Sjodt et al, we assumed that too many changes would not have been appreciated by the journal. We have now included this paper in several sections.

2a. Concerning position of L61 in EcPBP2, I superimposed both structures to know the precise equivalences among E. coli and T. Thermophilus homologues. Indeed, L61 in E. coli corresponds to L43 in T. thermophilus. In the full-length TtPBP2:RodA complex this L43 is located at the interface with RodA in the so-called pedestal domain that Sjodt et al 2020 propose activates glycosyltransferase (GT) activity in RodA. Interestingly, Sjodt et al 2020 have done the equivalent mutant than Liu et al; that is L43R (L61R in E. coli) but some of the conclusions seem to be different: while they confirm the PBP2:RodA complex is formed with the mutant (in agreement with Liu et al) they provide in vitro experimental information that the "GT activity was reduced when Arg was introduced at residue L43". I think it is opposed to one of the sections by Liu et al manuscript ("PBP2L61R stays in the off state and activates RodA") (By the way, I am not sure if these data in Sjodt et al 2020 are also in contradiction with that reported by Rohs et al PLOS Gen 2018). This point should be discussed, as maybe with the 3D structure in face, some EPR results could be better understood and/or provide new insights on this matter.
2b. In same sense, the crystal structure of TtPBP2:RodA complex (PDB code 6PL6) revealed an unknown density in between the V-shaped pedestal domain that could correspond to some remnants of MreC. Indeed, creation of a mutant harboring a disulfide bridge linking both sides of the PBP2 pedestal domain (thus closing the central cavity) resulted in severe morphological defects, probably as a result of a reduced interaction with MreC. How FRET results agree with these data? Could you interpret MreC interaction with PBP2 in the light of this site? Are there other potential explanations?

Interestingly, the manuscript on the RodA/PBP2 crystal structure by Sjodt et al and the manuscript on the L61R mutant by Rohs et al have at least a number of authors in common. In the first paper PBP2L61R is claimed to inhibit the TGase activity of RodA and in the second it is claimed that the TGase activity of RodA is increased. If the owners of the data already do not agree themselves on their data, how should we decide which data reflect the reality?

The data of Rohs et al, show that the Rod system produces more PG and has a higher turnover if PBP2L61R is present. They also show in vitro that longer and more glycan stands are produced. They show that the MreB filaments are shorter, which results in more rotating and PG synthesizing complexes in the cell. Overall this seems to be in agreement with our observation that the glycan strands are on average longer in the isolated PG of the PBP2L61R mutants and that the cells are longer and thinner as also observed by Rohs. Although Sjod et al show in vitro that less PG is synthesized by the RodA/PBP2L61R combination, we cannot find a good argument why we should ignore all the data of Rohs in favor of the single experiment by Sjodt.

As mentioned by the reviewer, the closed V-shaped pedestal domain of PBP2 likely abolishes the interaction between PBP2 and MreC. Actually, our FRET results are in agreement with this. We have also shown that when the interaction between MreC and PBP2 was prevented by MreD, the MreC induced conformational change of RodA-PBP2 was also abolished. Once the MreC-PBP2 interaction was reinstated by removing the cytoplasmic domain of PBP2 (^{MalFNT}PBP), which prevents the binding of MreD, the MreC induced conformational change in the PBP2-RodA complex was also restored. However, we are very hesitant to describe our FRET data in terms of specific structural changes. We can only measure that two fluorescence proteins get closer to each other, which indicates conformations changes in the proteins they are fused to.

At this moment no data are available that show which amino acids should be mutated to prevent the activation of RodA by PBP2.The closed V-shapd PBP2 protein is inactive and not able to bind MreC (Sjodt et al., 2020 and Contreras et al 2017).

Reviewer #3: The revised version of the manuscript by Liu et al. has failed to address the important inconsistencies pointed out in my review that call into question the validity of their main conclusions.

The authors opted not to carry out an obvious and important control – the effect of MreD overexpression on the PBP2-RodA FRET signal. The goal of this control is to rule out the possibility that reduction in the PBP2-RodA FRET signal observed with MreC could be a general response to perturbing the stoichiometry of the Rod complex. The authors argued that "Because of the lack of strong effects on the morphology of the cells by MreD,

we do not expect an effect of MreD on the interaction between RodA and PBP2". By the same logic, we would not expect MreC to have an effect on the PBP2-RodA interaction either.

We have now performed the requested experiment in which the FRET pair PBP2+RodA are expressed in the presence of MreD. We do not see a difference in the interaction between PBP2 and RodA in the presence or absence of MreD. This information has been added to the manuscript as follows.

"Expression of MreD without MreC also did not affect the interaction between RodA and PBP2 as their EfA value in the presence of a third plasmid expressing MreD was 13.3 ± 2.9% (n= 9), which was not significant different from their EfA of 11.8 ± 1.4% (n=3) in the presence of the third, empty plasmid (Supplementary Fig. 4d)."

The second important point that was not addressed in any substantial way is the unexpected FRET behavior of the PBP2L61R mutant. This is a mutant that activates the Rod system in the absence of MreC, thus strongly suggesting that it makes the PBP2-RodA complex adopt the same conformation induced by MreC. Thus, the FRET behaviour of this mutant should mimic the FRET behavior in response to MreC. If this expectation is not borne out by the data I would be very worried about the validity of my hypothesis (low FRET = active conformation). Instead, the authors insist in explaining this unexpected result with convoluted and improbable reasoning. They argue that the PBP2L61R-RodA complex conformation need not be the same as the MreC-activated PBP2-RodA conformation because the mutation only stimulates the glycosyltransferase and not the transpeptidase activity of the complex. This seems to be based on the assumption that MreC stimulates both the glycosyltransferase and transpeptidase of PBP2-RodA, but, as far as I can tell, there is no data to support this. In fact, the observation that mutations in RodA can also bypass MreC led to the model that Rod complex activation occurs primarily through activation of the glycosyltransferase activity of RodA (Rohs et al., 2018). The recently published structure of the PBP2-RodA complex seems to also support this model (Sjodt et al., 2020).

We agree with the reviewer that no data are available that show that MreC also activated the TPase activity of PBP2. Our data also suggest that L61R is only activating the TGase of RodA and does not change the TPase activity of PBP2. We have removed wording about the on and off state of PBP2 in relation to L61R and simply conclude that L61R activates RodA but does not change the interaction of RodA and PBP2 as measured by FRET. Since no conformational changes are observed by FRET for the L61R mutant and mecillinam still reduces the FRET efficiency for the PBP2L61R RodA complex, we think that the MreC induced conformational change that is elevated by MreD is different from the activation due to the L61R mutation. This is also supported by the only partial complementation of the defect caused by the absence of MreC in the L61R mutant.

As stated in my initial review, I suspect it will not be simple to sort out what is going on with PBP2L61R. However, there are relatively simple experiments that could have been be done to increase our confidence that the FRET data is really telling us what the authors claim. In addition to the MreD control, it would be possible to test mutations that prevent the interaction between PBP2 and MreC (Contreras-Martel et al., 2017) as well as mutations that prevent the allosteric activation of RodA by PBP2 (Sjodt et al., 2020). If these mutants behave as expected (being insenstive to MreC in a 3 plasmid experient) this would substantially strengthen the conclusions of the manuscript.

Using the information of (Contreras-Martel et al., 2017), mutation of *H pylori* MreC Phe169, Phe182,and Tyr222 abolishes the interaction of MreC with PBP2. Alignment of *H. pylori* MreC with *E. coli* MreC, shows that the corresponding residues in *E. coli* do not exist. We could mutate *E. coli* MreC residues in the vicinity of the corresponding *H. pylori* residues and then isolate the protein to do circular dichroism to show that it is correctly folded and then do a three plasmid experiment, hoping the we have mutated the right residues and do not see an change in the FRET efficiency of PBP2 and RodA anymore. With all due respect, we think that this is not a reasonable request.

Actually, if the reviewer asks for evidences that "preventing the interaction between PBP2 and MreC will abolish the reduction of RodA-PBP2 FRET in the presence of MreC", we think our current manuscript has shown sufficient and convincing data to support this. We have shown that expression of MreD prevents the interaction between PBP2 and MreC as well as the observed reduction in FRET efficiency of the PBP2-RodA complex by MreC (Fig. 4c). MreD itself did not reduce the RodA-PBP2 FRET efficiency and was also shown to interact with PBP2. Moreover, we have also shown that when interaction with MreD is prevented using the PBP2 domain swap mutant ^{MalFNT}PBP2, MreC is again reducing the FRET efficiency of ^{MalFNT}PBP2-RodA. All these reduced/restored FRET results strongly suggest that the reduction of RodA-PBP2 FRET is sensitive enough and specific to MreC,

At this moment no data are available that show which amino acids should be mutated to prevent the activation of RodA by PBP2.The closed V-shapd PBP2 protein is inactive and not able to bind MreC (Sjodt et al., 2020 and Contreras et al 2017). Since we know, based on our FRET data, that RodA-PBP2 is not changing its conformation in the absence of MreC and based on Sjodt's data that the PBP2 protein is inactive with the disulfide bond, the predicted outcome of a FRET experiment would be. 1) closure of the V-shape changes the RodA-PBP2 conformation and changes the FRET efficiency, MreC does not further change it as we know it cannot bind. 2) closure of the V-shape does not change the RodA-PBP2 conformation and does not changes the FRET efficiency, MreC does not further change it as we know it cannot bind. We do not see that this experiment would give more information than the information we have already provided.

We tried to combine the L61R mutant with MreC and MreCD in the three plasmid FRET system as stated in the manuscript, but most times the cells lost the L61 construct indicating that this combination is toxic. Apparently L61R activates RodAPBP2 without the need of MreC, but binding of MreC might make the activation more efficient causing uncontrolled PG synthesis and death of the cells. The presence of MreC is not identical to the L61R mutant as the former gives a conformational change and the latter not (at least not one that can be measured by FRET).

We have added text (highlighted in yellow) in the discussion to point out these various issues.

Minor points:

l 253. In contrast to what is stated, Supplementary Figure 5b shows that the co-overexpression of MreC and D leads to the same aberrant cell shape as the overexpression of the individual proteins.

We changed the text as follows:

"During our study, we noticed that overexpression of MreC caused morphological defects of the wild-type strain grown in rich medium, decreasing the length and increasing the diameter of E. coli cells (Fig. 4e and Supplementary Fig. 5). Interestingly, the co-expression of MreD together with MreC suppressed at least the decrease in length "

l 268. ... likely attributed ...

Changed as suggested.

Have all data underlying the figures and results presented in the manuscript been provided?
Large-scale datasets should be made available via a public repository as described in the *PLOS Genetics* data availability policy, and numerical data that underlies graphs or summary statistics should be provided in spreadsheet form as supporting information.

Reviewer #1: Yes

Reviewer #2: Yes

Reviewer #3: Yes

PLOS authors have the option to publish the peer review history of their article (what does this mean?). If published, this will include your full peer review and any attached files.

If you choose "no", your identity will remain anonymous but your review may still be made public.

Do you want your identity to be public for this peer review? For information about this choice, including consent withdrawal, please see our Privacy Policy.

Reviewer #1: No

Reviewer #2: No

Reviewer #3: No
